# Individual and community level factors associated with modern contraceptive utilization among married women in the emerging region of Ethiopia: a multilevel mixed effects analysis of the 2019 Ethiopia Mini-Demographic and health survey

**DOI:** 10.1186/s12905-023-02822-1

**Published:** 2023-12-07

**Authors:** Natnael Kebede, Bereket Kefale, Muluken Yigezu, Eyob Ketema Bogale, Amare Zewdie, Yitbarek Wasihun, Metadel Adane

**Affiliations:** 1 Department of Health Promotion, School of Public Health, College of Medicine and Health Sciences, Wollo University, Dessie, Ethiopia; 2 Department of Reproductive and Family Health, School of Public Health, College of Medicine and Health Sciences, Wollo University, Dessie, Ethiopia; 3 Department of Public Health College of Medicine & Health Science, Dire Dawa, Dire Dawa University, Dire Dawa, Ethiopia; 4 Department of Health Promotion, College of Medicine Health Sciences, Bahir Dar University, Bahir Dar, Ethiopia; 5 Department of Public Health, College of Medicine and Health Science, Wolkite University, Wolkite 07, Ethiopia; 6 Department of Environmental Health, College of Medicine and Health Sciences, Wollo University, Dessie, Ethiopia

**Keywords:** Modern contraceptive utilization, A multilevel mixed effects analysis, Married women, Ethiopia

## Abstract

**Background:**

A demonstrated technique to enhance reproductive health and economic progress is through ensuring that family planning services are accessible universally. Those studies that used Ethiopia Demographic and Health Survey (EDHS) data did not assess individual and community-level factors in contraceptive utilization. Thus, the study employs a multilevel mixed effects analysis approach, which allows for the examination of individual and community-level factors that influence contraceptive utilization.

**Methods:**

This study analyzed the 2019 Ethiopia Min Demographic and Health Survey datasets. A total of 1916 married women in the 2019 surveys were included in the analysis. The data were analyzed using Stata version 17.0. The data were analyzed using Multi-level mixed-effect logistic regression to identify the individual and community-level factors associated with modern contraceptive utilization. An adjusted odds ratio with a 95% confidence interval was used to.

Show the strength and direction of the association and statistical significance was declared at a *P* value less than 0.05.

**Results:**

Factors significantly associated with modern contraceptive utilization were; Muslim and protestant followers [AOR = 0.31, 95% CI: (0.134, 0.714)] and [AOR = 0.35, 95% CI: (0.173, 0.691)] respectively, women with no education (OR = 0.46; 95% CI: 0.293, 0.710), those women who belong to the poor and middle wealth of household [AOR = 0.35, 95% CI: (0.237, 0.527)] and [AOR = 0.56, 95% CI: (0.347, 0.919)] respectively, women who had one to five and greater than or equal to six living children [AOR = 11.36, 95% CI:(2.119, 60.918)] and [AOR = 7.44, 95% CI:(1.437, 38.547)]respectively, Women in clusters poor wealth status [AOR = 0.40, 95% CI: (0.183, 0.875)] and women who belong to the Somali region [AOR = 0.20, 95% CI: (0.0.070, 0.506)].

**Conclusion:**

The study revealed that both individual and community-level factors determined modern contraceptive utilization. At the individual level, the religion of women, educational status, the wealth of the household, and the total number of living children were significantly associated with modern contraceptive utilization. At community-level factors, community wealth status and belonging to the Somali region were significantly associated with modern contraceptive utilization. The findings suggest that interventions aimed at increasing modern contraceptive utilization should target women with lower levels of education, those living in households with lower wealth, and those with larger families. Additionally, efforts should be made to improve access to modern contraceptives in communities with lower wealth status and in regions where traditional beliefs may hinder their use.

## Introduction

Family planning has been associated with several benefits such as a reduction in maternal and infant mortality [1, 2]. Globally, 44.2% of women of reproductive age (15–49 years) used a contraceptive method in 2019 [3]. Globally, contraceptive use among women of reproductive potential who are married or in a union has risen from 55% in 1990 to 63% in 2020 [4]. Contraceptive use among married/cohabiting women in sub-Saharan Africa increased from 13 to 33% over the same period and remains the lowest in the region [4]. In Ethiopia, modern contraceptive use rose from 6% in 2000 to 40% in 2019. However, this trend varies widely across regions, stagnating in the emerging regions of Ethiopia, where contraceptive use has increased by only 5% over 20 years [5, 6].

Family planning is one of the proven strategies to reduce maternal and child morbidity and mortality. It improves maternal health by reducing the number of pregnancies [7, 8] and avoiding unintended and closely spaced pregnancies [9]. Furthermore, if all unmet needs for modern contraception in developing regions were met, unintended pregnancies, unplanned births, and abortions would be reduced by about three-quarters. Comprehensive coverage of the unmet need for modern contraceptives would avert an estimated 76,000 maternal deaths per year [10]. Family planning also reduces child morbidity and mortality by avoiding too many and ill-timed pregnancies [11, 12].

The use of modern contraceptives is related to many factors. This includes; age [13, 14], marital status [14–16], religion [14, 17, 18], residence [19, 20], educational status [14, 18–23], wealth index [14, 19, 20, 23], media exposure [20], discussion on family planning with a partner [16, 17, 24–26], knowledge on modern contraceptive methods [13, 21, 27], ever use of a modern contraceptive method [15, 17], number of alive children [18–20], and the desired number of children [13, 19, 21].

Investment in family planning is critical to achieving most of the Sustainable Development Goals (SDGs), mainly SDG 3 and SDG 5 [28]. Because of this, several governmental and non-governmental organizations have made enormous efforts to increase access to family planning services. The Government of Ethiopia has set a target of increasing contraceptive prevalence to 55% by 2020 [29]. However, only 41% of married women used birth control in 2019 indicating a major challenge to access contraceptives.

The emerging regions of Ethiopia are characterized by cultural, social, and economic diversity, which may influence the utilization of modern contraceptives. Therefore, understanding the determinants that drive the use of modern contraceptives in these regions is important to tailor interventions that target specific issues related to contraceptive utilization. Assessing the individual and community-level factors of modern contraceptive utilization for the change is vital to designing appropriate interventions to increase family planning service utilization. Most studies conducted on modern contraceptive utilization were only localized to certain settings and their sample size was too small [17, 21, 25, 26]. Moreover, those studies that used Ethiopia Demographic and Health Survey (EDHS) data did not assess individual and community-level factors in contraceptive utilization [30]. Thus, the study employs a multilevel mixed effects analysis approach, which allows for the examination of individual and community-level factors that influence contraceptive utilization. This approach provides a more nuanced understanding of the complex dynamics related to contraceptive utilization, which is critical for the design of targeted interventions at both levels.

## Material and methods

### Study area and data source

The study was conducted in emerging regions of Ethiopia, which are Afar, Somali, Benshangul Gumz, and Gambela regions. Emerging regions in Ethiopia refer to areas that have relatively low levels of socio-economic development and infrastructure compared to other regions in the country. These regions are identified based on national development criteria and include Afar, Somali, Benshangul Gumz, and Gambela [31]. Ethiopia is one of the Sub-Saharan African countries. This study used 2019 MEDHS data sets, which were collected by the Central Statistical Agency (CSA) in coordination with the Federal Minister of Health (FMOH) and the Ethiopia Public Health Institute (EPHI). The secondary data for this analysis were obtained from mini-Edhs of 2019 that were found at the DHS portal (https://DHSprogram.com/data/dataset/Ethiopia_Interim-DHS_2019).

The 2019 EMDHS sample was stratified and selected in two stages. Each region was stratified into urban and rural areas, yielding 21 sampling strata. Each cluster contained 30 households. Households were systematically sampled using random selection. All women of reproductive age who were either usual members of the selected households or who slept in the household the night before the survey were eligible for interview. The EDHS is representative nationally, regionally, and by urban-rural residents. The survey was conducted from January through April 2019 in all four regions of Ethiopia. Since the outcome variable for this study was modern contraceptive utilization a total of 1, 916 married women in 2019 MEDHS were included for analysis.

### Variable measurement

The dependent variable of modern contraceptive utilization was classified dichotomously as “Yes/No”. Married women who used a modern contraceptive method during the time of the interview were categorized as “Yes” and those who did not use a modern contraceptive method during the time of the interview were categorized as “No”. Modern contraceptive methods, which include intrauterine devices (IUDs), hormonal methods (such as pills, patches, injections, and vaginal rings), barrier methods (such as condoms and diaphragms), and sterilization (such as tubal ligation and vasectomy) [32].

#### Individual-level variables

Maternal age, educational status, religion, wealth status, and the number of children.

#### Community-level variable

Region, place of residence, community education, and community wealth status.

### Data quality, processing, and analysis

The data used in the current research was collected through the Ethiopian Demographic Health Survey (DHS), which was conducted by the Central Statistical Agency (CSA) in coordination with the Federal Minister of Health (FMOH). The DHS study followed strict ethical procedures, including the Helsinki Declaration, to ensure the safety and confidentiality of the participants.

To ensure consistency and completeness, data cleaning was performed before recording, labeling, and exploratory analysis using Stata/SE version 17.0. Frequency distributions were presented in tables, graphs, and text using descriptive statistics.

A sample weight was used to compensate for potential disparities in geographical strata selection and non-responses. Multilevel analysis was conducted after checking that the data were eligible for multilevel analysis which means Intra-cluster Correlation Coefficient (ICC) greater than 10% (ICC = 20.53%). Since DHS data are hierarchical, i.e. individuals (level 1) were nested within communities (level 2), a two-level mixed-effects logistic regression model was fitted to estimate both independent (fixed) effects of the explanatory variables and community-level random effects on modern contraceptive utilization. The log of the probability of modern contraceptive utilization was modeled using a two-level multilevel model.

An initial bivariable multilevel logistic regression was performed, selecting variables with a *p*-value < 0.25 before creating three models (models 1–3). Four stages were then conducted: Model 0 (empty or null model without explanatory variables), Model 1 (only individual-level factors), Model 2 (only community factors), and Model 3 (both individual and community-level factors). The measures of association (fixed-effects) estimate the associations between the likelihood of women to modern contraceptive utilization and various explanatory variables were expressed as Adjusted Odds Ratio (AOR) with their 95% confidence level. A variable in which its p-value < 0.05 was used to declare statistical significance. The measures of variation (random effects) were reported using ICC, Median Odds Ratio (MOR), and proportional change in variance (PCV) to measure the variation between clusters.

The ICC shows the variation in modern contraceptive utilization for married reproductive women due to community characteristics. The higher the ICC, the more relevant the community characteristics for understanding individual variation in modern contraceptive utilization for married reproductive women.

MOR is defined as the median value of the odds ratio between the area at the highest risk and the area at the lowest risk when randomly picking out two areas. In this study, MOR shows the extent to which the individual probability of modern contraceptive utilization for married reproductive women is determined by residential area. PCV measures the total variation attributed to individual-level factors and area-level factors in the multilevel model.

The presence of multicollinearity was checked among independent variables using standard error at the cutoff point of ±2 and there was no multicollinearity. The log-likelihood test was used to estimate the goodness of fit of the adjusted final model in comparison to the preceding models (individual and community-level model adjustments).

## Result

### Socio-demographic characteristics of respondents

The total number of married women included in the analysis was 1916. The mean age of respondents was 29.18 years (±0.18) in 2019. Almost three-fourths (72.01%) of women were not formally educated. Concerning the wealth status of the household, 312(70.96%) women were poor and 378(85.90%) women were Muslim followers.

The unit of analysis for the characteristics of community-level factors was the cluster. For this study, we include a cluster of 205 married women. Similar to the higher rural than urban proportion of the population in Ethiopia, 67.85% of the clusters were in rural areas. In this study, 283(64.50%) of the clusters were Somali regions, while the rest were either other three regions. The current study attempted to form community-level factors by aggregating values of different individual characteristics. Accordingly, 58.5% of the clusters had lower levels of wealth status, based on the aggregate values derived from the EDHS data on the wealth index and modern contraceptive utilization. Almost 97% of the clusters had lower than secondary school educational attainment (Tables 1, 2, and 3).

**Table 1:** Distribution characteristics of participants in 2019 EMDHS

Category MEDHS, *n* = 1916	Unweight n (%)	Weighted n (%)
**Age**		
15–24 years	563(29.3)	122(27.72)
25–34 years	814(42.48)	189(42.95)
35–49 years	539(28.13)	129 (29.33)
**Residence**		
Urban	338(17.64)	141(32.15)
Rural	1578(82.36)	2987(67.85)
**Religion**		
Orthodox	327(17.07)	37(8.46)
Muslim	1230 (64.20)	378(85.90)
Protestant	319 (16.65)	22(5.09)
Other^a^	40(2.09)	2(0.55)
**Educational status**		
Not educated	1150 (60.02)	317(72.01)
Primary	564 (29.44)	92(20.82)
Secondary and above	202 (10.54)	32(7.17)
**Wealth status**		
Poor	1221(63.73)	312(70.96)
Middle	248(12.94)	34(7.74)
Rich	447(23.33)	94(21.30)

^a^Catholic and traditional religion follower

**Table 2:** Distribution characteristics of participants in 2019 EMDHS

Category MEDHS, *n* = 1916	Unweight n (%)	Weightedn (%)
Regions		
Afar	482 (25.16)	64(14.59)
Somali	446 (23.28)	283(64.50)
Benshangul Gumz	530(27.66)	67(15.19)
Gambela	458 (23.90)	25(5.72)
**Community wealth status**		
Lower than middle and rich	921(48.07)	258(58.50)
Higher than middle and rich	995(51.93)	183(41.50)

**Table 3:** Obstetric characteristics of participants in 2019 EMDHS

**Ever give birth**		
Yes	1716 (89.56)	392(89.07)
No	200(10.43)	48(10.93)
**Number of living children**		
0	227(11.84)	54(12.22)
1–5	1290 (67.33)	261(59.32)
≥6	399 (20.82)	125(28.47)

### Modern contraceptive utilization

According to the mini Ethiopia health survey report, the utilization of modern contraceptives among married women in 2019 was 21.97% (20.17, 23.88).

### Individual and community-level factors associated with modern contraceptive utilization (fixed-effects)

After adjusting for individual and community-level factors (model 3) religion of women, educational status, wealth of household, the total number of living children, community wealth status, and region were found to have a statistically significant association with Modern contraceptive utilization. Those Muslim and protestant women were 0.3 and 0.35 times less likely to use modern contraceptives as compared to Orthodox followers [AOR = 0.31, 95% CI: (0.134, 0.714)] and [AOR = 0.35, 95% CI: (0.173, 0.691)] respectively. The odds of modern contraceptive utilization were 0.5 times (OR = 0.46; 95% CI: 0.293, 0.710) lower among women with no education compared with those who attended secondary (or higher) school and above.

Those women who belong to poor and middle wealth of households were 65 and 43% less likely to use modern contraceptives as compared to the rich [AOR = 0.35, 95% CI: (0.237, 0.527)] and [AOR = 0.56, 95% CI: (0.347, 0.919)] respectively.

The odds of modern contraceptive utilization for women who had one to five and greater than or equal to six living children were, about 11 times and 7 times higher than those who had no children [AOR = 11.36, 95% CI:(2.119, 60.918)] and [AOR = 7.44, 95% CI:(1.437, 38.547)]respectively.

Women in clusters with higher relative poverty levels had a lower likelihood of using modern contraceptives [AOR = 0.40, 95% CI: (0.183, 0.875)] than women in clusters with middle and above wealth status levels. Finally, those women who belong to the Somali region were 80% less likely to use modern contraceptives than the Benshangul Gumz region [AOR = 0.20, 95% CI: (0.0.070, 0.506)] (Table 4).

**Table 4:** Multi-level mixed effect logistic regression on modern contraceptive utilization among married women in Ethiopia, 2019 EDHS dataset

Individual and community characteristics	Model 0	Model 1 AOR (95% CI)	Model 2 AOR(95%CI)	Model 3 AOR (95% CI)
**Age**				
15–24 years		1	1	1
25–34 years		0.99 (0.656, 1.489)		0.91 (0.617, 1.33)
35–49 years		0.78(0.500, 1.22)		0.54 (0.415, 0.981)
**Religion**				
Orthodox		1	1	1
Muslim		**0.14(0.084, 0.231)**		**0.31 (0.134, 0.714)**
Protestant		**0.29(0.156, 0.554)**		**0.35(0.173, 0 .696)**
Other*		**0.22(0.052, 0.935)**		0.26(0.056, 1.219)
**Educational status**				
Not educated		**0.44(0.281, 0.675)**		**0.46 (0.293, 0.710)**
Primary		1.08(0.742, 1.585)		0.84 (0.541, 1.303)
Secondary and above		1	1	1
**Wealth status**				
Poor		**0.29(0.182, 0.456)**		**0.35 (0.237, 0.527)**
Middle		0.89(0.538, 1.464)		**0.56 (0.347, 0.919)**
Rich		1	1	1
**Ever give birth**				
Yes		0.30(0.078, 1.168)		0.40 (0.095, 1.692)
No		1	1	1
**Number of living children**				
0		1	1	1
1–5		**15.80(2.78, 89.80)**		**11.36 (2.119, 60.918)**
≥ 6		**8.85(1.52 51.48)**		**7.44 (1.437, 38.547)**
**Place of residence**				
Urban			0.92(0.463, 1.825)	0.60 (0.286, 1.271)
Rural			1	1
**Region**				
Benshangul Gumz			1	1
Somali			**0.11(0.039, 0.295)**	**0.20 (0.070, 0 .506)**
Afar			**0.43(0.212, 0.874)**	0.60 (0.297 1.213)
Gambela			0.80(0.450, 1.417)	0.71 (0.389, 1.279)
**Community education status**				
Lower			1	1
Higher			0.89(0.449, 1.786)	0.52 (0.219, 1.221)
**Community wealth status**				
Lower			**0.23(0.103, 0.519)**	**0.40 (0.183, 0.875)**
Higher			1	1
**Random effects**				
Variance	**0.85**	0.53	0.23	0.11
The intra-cluster correlation coefficient (ICC) in %	**20.53**	13.87	6.53	3.33
Median odds ratio (MOR)	**2.40**	2.00	1.58	1.37
Explained variance (PCV %)	**Reference**	38	52	57
**Model fitness** Log-likelihood	− 153.69922	− 117.66345	− 120.1637	− 107.90524

### Random effect (a measure of variation)

The results of multilevel logistic regression for random effects showed that there was a significant variation in Modern contraceptive utilization across the clusters (Table 4). The correlation coefficient in the cluster shows that 20.53% of the variation in modern contraception is related to the factors at the community level. The complete model also showed that there is a statistically.

Significant variation in Modern contraceptive utilization across communities or clusters. The entire model explains about 57% of modern contraceptive utilization in clusters. MOR also confirmed that contraception is a factor attributed to the community level. The MOR for Modern contraceptive utilization was 2.4 times in the empty model which indicates that there are differences (clustering) between the communities. When all factors are added to the model, the unexplained community variation in Modern contraceptive utilization decreased to MOR of 1.37 times. This shows that if all factors are considered, the impact of clustering in the complete model is still statistically significant (Table 4).

## Discussion

This study aimed to identify the individual- and community-level factors of modern contraceptive utilization among married women in the emerging regions of Ethiopia. The proportion of modern contraceptive utilization among married women was 21.97% in 2019 MEDHS. This proportion of modern contraceptive utilization reflects different individual and community factors that affect modern contraceptive utilization. At the individual level, the religion of women, educational status, the wealth of the household, and the total number of living children were significantly associated with modern contraceptive utilization. At the community level, community wealth status and region were found to have a statistically significant association with modern contraceptive utilization. The differences in the use of contraceptive methods in different regions of Ethiopia can be attributed to several factors. Firstly, there may be variations in the availability and accessibility of family planning services across regions. Some regions may have better access to family planning services, while others may have limited access due to factors such as distance, infrastructure, and availability of trained health professionals.

In this study, women with no education were associated with modern contraceptive utilization. The finding of this study is in line with previous studies in Ethiopia [33], Tanzania [34], Ghana [35], and Nigeria [36]. It has been congruent that progress in education has a key role in shaping individuals’ perceptions and knowledge about modern contraceptives, which helps them in understanding misconceptions gap to modern contraception use. In addition, Education can promote decision-making capabilities and autonomy, increase the choice of economic freedom, and affect women’s current and future fertility plans [37]. Moreover, increased contraceptive uptake and fertility control were seen as having a higher level of education in women [38]. This may involve targeted education campaigns that focus on the benefits of family planning and dispel myths and misconceptions about contraceptive methods.

This study reveals that poor and middle wealth index quintiles were less likely to use modern contraceptive methods relative to the rich wealth category. A similar outcome is recognized in studies conducted in different African nations like Nigeria [39], Tanzania [40], Ghana [41], and Malawi [42]. Additionally, the influence of wealth status on modern contraceptive usage was also confirmed that women in higher wealth quintiles were significantly more likely to use postpartum family planning compared to those within the lowest wealth quintiles [42, 43]. Women from poor and middle-class households have limited access to healthcare services, education, and resources related to reproductive health. Since modern contraceptives are not easily available, affordable, or accessible to them, they may rely on traditional or less effective methods of birth control or none at all because of these factors they might not have the perceived behavioral control to utilize modern contraceptives [44] but this explanation is might not be appropriate for Ethiopia since contraceptive is freely given rather the general socio-cultural factors makes the difference. It suggests that efforts to increase access to family planning services and promote contraceptive use should prioritize reaching out to women from lower socioeconomic backgrounds.

The current study also showed that the religion of the woman was significantly associated with modern contraceptive utilization. Women who had been following Muslim and Protestant religions were less likely to utilize modern contraceptives. This is in line with the studies [18, 33, 45–48]. The main reason for this difference might be religious beliefs. Perhaps Orthodox followers are more open to using modern contraceptive methods than Muslim or Protestant women due to differences in religious teachings or attitudes towards contraception.

As indicated in this result, the number of living children was associated with modern contraceptive usage. The odds of modern contraceptive utilization for women who had one to five and greater than or equal to six living children were more likely to use modern contraceptives than those who had no children. This study is also consistent with the previous studies [49–52]. One possible explanation is that women who have already had one to five or six or more children may have a greater awareness of the challenges and responsibilities that come with raising a child and may be more likely to want to use modern contraceptives to prevent unintended pregnancies. Additionally, women who have had no children may not have as much experience with pregnancy and childbirth, and may not feel as comfortable using contraceptives. Another possible explanation is that women who have one to five or six or more children may have greater access to and knowledge about modern contraceptives through healthcare providers or community outreach programs, whereas women with no children may not have as many opportunities to learn about and gain access to these resources.

In contrast to this women who planned to have more children or those who perceived and desired to have more children were less likely to use modern contraceptive studies in Mali [53] and Indonesia [50]. The likely explanation for married women with a greater preferred number of children not utilizing contraception could be that children are cherished assets for future household-level economic prosperity. This may be advantageous in nations with low resources, as having more children may boost household income because more members of the home are likely to enter the labor force [54].

The residence region of women was also significantly associated with modern contraceptive utilization among married women in emerging regions of Ethiopia. There was an 80% decrease in utilized modern contraceptives in Somali regions compared to the Benshangul Gumz region. It is consistent with the results of previous studies in Ghana [35] and Nigeria [36]. This regional discrepancy might be due to there may be cultural or religious beliefs surrounding contraception that are more prevalent in Somali regions, leading to a lower uptake of modern contraceptives.

The strength of the current study includes the use of multilevel mixed effect analysis it accounts for the nested structure of data, where observations are nested within groups or clusters, allowing for the estimation of both within-group and between-group effects and the examination of individual and group-level predictors simultaneously. The study also has some limitations, it assumes that the effects of predictors are constant across all levels, which may not always be the case. It may be prone to bias if there is unobserved heterogeneity across groups or clusters.

## Conclusions

This study provides evidence that both individual and community-level factors are important determinants of modern contraceptive utilization. The findings highlight the significance of factors such as religion, education, household wealth, and number of living children at the individual level, and community wealth status and regional differences at the community level. These results suggest that targeted interventions aimed at addressing these specific determinants could be effective in increasing modern contraceptive utilization in the population. Moreover, this study provides a valuable contribution to the existing literature by specifying the differences between modern contraceptive utilization and general family planning utilization. Overall, these findings have important implications for policy and program planning in the field of reproductive health.

## Data Availability

All the necessary data are included in the manuscript. The detailed information was found within the EDHS report and the data set by requesting permission through the website www.measuredhs.com.

## Abbreviations

CSA: Central Statistical Agency
EDHS: Ethiopia Demographic, and Health Survey
EMDHS: Ethiopia Min Demographic, and Health Survey
EPHI: Ethiopia Public Health Institute
FMOH: Federal Ministry of Health
SDG: Sustainable Development Goals
